# Gut microbiota composition and metabolic characteristics in patients with Craniopharyngioma

**DOI:** 10.1186/s12885-024-12283-w

**Published:** 2024-04-25

**Authors:** Chunhui Liu, Fangzheng Liu, Ding Nie, Youchao Xiao, Wentao Wu, Yanfei Jia, Lu Jin, Ning Qiao, Kefan Cai, Siming Ru, Xin Liu, Yifan Song, Jintian Xu, Lei Cao, Songbai Gui

**Affiliations:** 1 Department of Neurosurgery, Beijing Tiantan Hospital, Capital Medical University, Beijing, 100071 China; 2 Beijing Neurosurgical Institute, Capital Medical University, Beijing, 100071 China

**Keywords:** Craniopharyngioma, Gut microbiota, Hypothalamic-pituitary-adrenal axis, Gut-brain axis

## Abstract

**Background:**

Emerging evidence suggests that the gut microbiota is associated with various intracranial neoplastic diseases. It has been observed that alterations in the gut microbiota are present in gliomas, meningiomas, and pituitary neuroendocrine tumors (Pit-NETs). However, the correlation between gut microbiota and craniopharyngioma (CP), a rare embryonic malformation tumor in the sellar region, has not been previously mentioned. Consequently, this study aimed to investigate the gut microbiota composition and metabolic patterns in CP patients, with the goal of identifying potential therapeutic approaches.

**Methods:**

We enrolled 15 medication-free and non-operated patients with CP and 15 healthy controls (HCs), conducting sequential metagenomic and metabolomic analyses on fecal samples to investigate changes in the gut microbiota of CP patients.

**Results:**

The composition of gut microbiota in patients with CP compared to HCs show significant discrepancies at both the genus and species levels. The CP group exhibits greater species diversity. And the metabolic patterns between the two groups vary markedly.

**Conclusions:**

The gut microbiota composition and metabolic patterns in patients with CP differ significantly from the healthy population, presenting potential new therapeutic opportunities.

## Introduction

Craniopharyngiomas (CPs) are rare primary brain tumors that originate from remnants of the craniopharyngeal duct epithelium [1]. The incidence of this rare tumor is approximately 0.19 per 100,000 persons, constituting 1.2–4.6% of all intracranial tumors [1, 2]. Despite their rarity, their close anatomical proximity to critical structures such as the hypothalamus, pituitary gland, and optic chiasm can lead to a poor prognosis.

Surgical resection remains the primary treatment for CPs, and the extended endoscopic endonasal approach (EEEA) has emerged as a reliable method for achieving gross-total resection (GTR) in the past decade [3–5]. Detecting CPs preoperatively is challenging, as patients typically present no symptoms or signs during the initial stages of tumor development. Furthermore, individuals diagnosed with CPs necessitate long-term follow-up and hormone replacement therapy post-surgery. Currently, no effective noninvasive therapy has been identified for CPs. Therefore, there is a need for a profound understanding of the characteristics of the disease and exploration of potential treatments.

Due to advancements in DNA sequencing technology and novel bioinformatics tools, the gut microbiota is now recognized as a crucial factor in human health, particularly in brain health. The gut microbiota, comprising *fungi*, *bacteria*, *bacteriophages*, *archaea*, *protozoa*, and *viruses*, influences hosts’ neuronal function through neurotransmitters, neuroactive compounds or other substances [6–9]. Moreover, the gut microbiota can interact with the hosts’ hypothalamic-pituitary-adrenal (HPA) axis by altering the individual’s neuroendocrine system [10, 11]. Like gliomas, pituitary neuroendocrine tumors (Pit-NETs), and other intracranial disorders, patients with CP may experience changes in the composition and metabolism of their gut microbiota, which could further impact the course of their disease. Recent studies reveal a notable correlation between gut microbiota and tumor progression in the sellar region [12–14]. Accordingly, our research focuses on the relationship between CPs and gut microbiota.

To delve deeper, we recruited 15 medication-free and non-operated patients with CP and 15 healthy controls (HCs). Our approach entailed sequential metagenomic and metabolomic analysis of fecal samples to explore changes in the gut microbiota associated with CPs.

## Materials and methods

### Clinical cohort

We recruited 15 patients with CP and 15 HCs from Beijing Tiantan Hospital of Capital Medical University. The collected cases were diagnosed based on the tumor classification criteria of the World Health Organization. Only patients definitively diagnosed with craniopharyngioma via postoperative pathological examination were included. Patients who had used antibiotics or gut microecological agents within 2 weeks, had a history of other diseases, smoking, alcohol abuse, or had a history of chronic digestive disease were excluded. For the HCs, we applied the same selection criteria and additionally matched them to the patient group in terms of gender and age. Upon admission, both patients with CP and HCs were provided with a standardized diet to minimize dietary differences. The study was approved by the Ethics Committee of Beijing Tiantan Hospital of Capital Medical University, and was conducted in accordance with the Declaration of Helsinki. All participants provided written consent for the use of their samples.

### Sample collection and metagenomic sequencing

Preoperative fecal samples were collected from all patients, and comparable samples were obtained from 15 healthy adults, matched by age and gender. These fecal samples were promptly sealed in a disposable sterile collection tube and stored at − 20 °C immediately after collection, then transferred to a − 80 °C freezer within 2 hours for long-term preservation.

Sequencing of the samples was conducted at Novozymes Bioinformatics Co, LTD. DNA was extracted using the magnetic soil and stool DNA kit (DP210831, TIANGEN BIOTECH, CHINA). Then, 1 μg of genomic DNA was fragmented into 350 bp pieces using Covaris ultrasonic fragmentation system for gene library construction. Initial quantification was performed using the Qubit 2.0 fluorometer, followed by dilution of the library to a concentration of 2 ng/μl. Then, the libraries underwent quality assessment, with those having an effective concentration > 3 nM deemed suitable for Illumina PE150 sequencing (involving 150 bp paired-end reads).

The metagenomic analysis of the CP group and HC group generated an average of 7897.6227 ± 2292.76890 Mb and 7392.1973 ± 126.22192 Mb of raw data, respectively (*p* = 0.401, *t*-test). Raw data were preprocessed with Readfq to obtain clean data for analysis. Reads were filtered out if they had low-quality bases (threshold ≤38) over 40 bp, N bases reaching 10 bp, or overlaps with adapters exceed 15 bp. The resulting clean data were assembled and analyzed using MEGAHIT software. And Scaftigs without N is obtained by breaking the resulted Scaffolds from the N junction.

MetaGeneMark is used to perform ORF prediction for Scaftigs (≥ 500 bp) of each sample, and the information with a length less than 100 nt in the prediction results is filtered out. For the ORF prediction results, CD-HIT software is used to eliminate redundancy and obtain the non-redundant initial gene catalogue. Clean Data is aligned to the initial gene catalog using Bowtie2 to obtain the final gene catalog (Unigenes) used for subsequent analysis.

DIAMOND software is used for alignment of Unigenes sequences with those of *bacteria*, *fungi*, *archaea*, and *viruses* extracted from NCBI’s NR database. The alignment results with evalue ≤ min. Evalue ^*^10 is selected. LCA algorithm is adopted to determine the species annotation information of the sequence since each sequence may have multiple alignment results.

Given the extensive information on metabolic pathways and biochemical processes for microbial communities provided by the KEGG database, it was selected as the preferred database for metagenomic research endeavors. The alignment of Unigenes with the KEGG database is similarly performed using DIAMOND software. The Best Blast Hit results from the alignment of each sequence are selected for subsequent analysis. According to the alignment results, the relative abundance at different functional levels is calculated.

### Untargeted metabolomics

A 100 mg fecal sample was placed in an EP tube, to which 500 μL of 80% methanol in water was added. The mixture was vortexed, shaken, and left to stand in an ice bath for 5 minutes, followed by centrifugation at 15,000 g for 20 minutes at 4 °C. The supernatant was then diluted with mass spectrometry-grade water to achieve a methanol concentration of 53% and centrifuged again for 20 minutes under identical conditions to collect the supernatant. Additionally, equal volumes from each experimental sample were combined to prepare a quality control sample. A 53% methanol solution was used as the blank sample.

Ultra-High-Performance Liquid Chromatography coupled with Tandem Mass Spectrometry (UHPLC-MS/MS) analyses were performed on a Vanquish UHPLC system (ThermoFisher, Germany) with an Orbitrap Q Exactive™ HF or HF-X mass spectrometer (ThermoFisher, Germany) in Novogene Co., Ltd. (Beijing, China). The separation was conducted on a Hypersil Gold column (100 × 2.1 mm, 1.9 μm) using a 12-minute linear gradient at a flow rate of 0.2 mL/min. For positive ion mode, the eluents were 0.1% formic acid in water (A) and methanol (B), whereas for negative ion mode, 5 mM ammonium acetate at pH 9.0 (A) and methanol (B) were used. The mass spectrometer operated in both positive and negative polarity modes, with parameters set to a spray voltage of 3.5 kV, capillary temperature at 320 °C, sheath gas flow at 35 psi, auxiliary gas flow at 10 L/min, S-lens RF level of 60, and auxiliary gas heater temperature at 350 °C.

Raw data were processed using Compound Discoverer 3.3 software to perform peak alignment, peak picking, and quantitation for each metabolite. The main parameters were set as follows: peak area was corrected with the first QC, actual mass tolerance, 5 ppm; signal intensity tolerance, 30%; and minimum intensity, et al. Then, peak intensities were normalized to the total spectral intensity to predict the molecular formula followed by matching with the mzCloud, mzVault, and Masslist databases. Metabolites were identified and relatively quantified after standardized processing, and annotations were performed using the KEGG, HMDB, and LIPIDMaps databases.

The quantitative results for differential metabolites and metabolic data from each comparative pair were then matched with the relative abundance values of differential genera at the corresponding genus level for analysis.

### Statistical analysis

The DIAMOND software facilitated the comparison of Unigenes against sequences from the NCBI’s NR database, with species annotation determined using the LCA algorithm. Analyses of PCoA, NMDS, and Anosim were performed using the ade4 and vegan packages in R software (version 2.15.3). The LEfSe software was employed to evaluate the differential abundance of taxa between two groups.

In multivariate statistical analysis, the metaX software transformed the data for Partial Least Squares Discriminant Analysis (PLS-DA), acquiring Variable importance in the projection (VIP values) for each metabolite. Univariate analysis involved calculating the statistical significance (*p*-value) and log fold change (FC) for each metabolite between the groups, using T-tests. The standard criteria for differential metabolite screening were set as VIP > 1, *p*-value < 0.05, and FC > 2 or FC < 0.5.

## Results

### Clinical characteristics of subjects

We enrolled 15 patients with CP and 15 HCs in the study. The average age of CP patients was 46.07 ± 13.93 years, and HCs averaged 48.07 ± 16.09 years. And the average Body Mass Index (BMI) of two groups was 26.37 ± 4.62 and 24.84 ± 2.15. No significant differences in age, gender or BMI were observed between the patients and the HCs (*p* = 0.719, *p* > 0.99, *p* = 0.259). The relevant clinical data for each individual is presented in Table 1.

**Table 1:** Clinical characteristics of subjects

	Total (*n* = 30)	CP (*n* = 15)	HC (*n* = 15)	*p* value
Gender, *n* (%)				> 0.99
Female	13 (43)	6 (40)	7 (47)	
Male	17 (57)	9 (60)	8 (53)	
Age, Mean ± SD	47.07 ± 14.82	46.07 ± 13.93	48.07 ± 16.09	0.719
BMI, Mean ± SD	25.60 ± 3.62	26.37 ± 4.62	24.84 ± 2.15	0.259

*CP* Craniopharyngioma, *HC* healthy control, *BMI* Body Mass Index

### Changes in gut microbiome composition in patients with CP

**Fig. 1:**
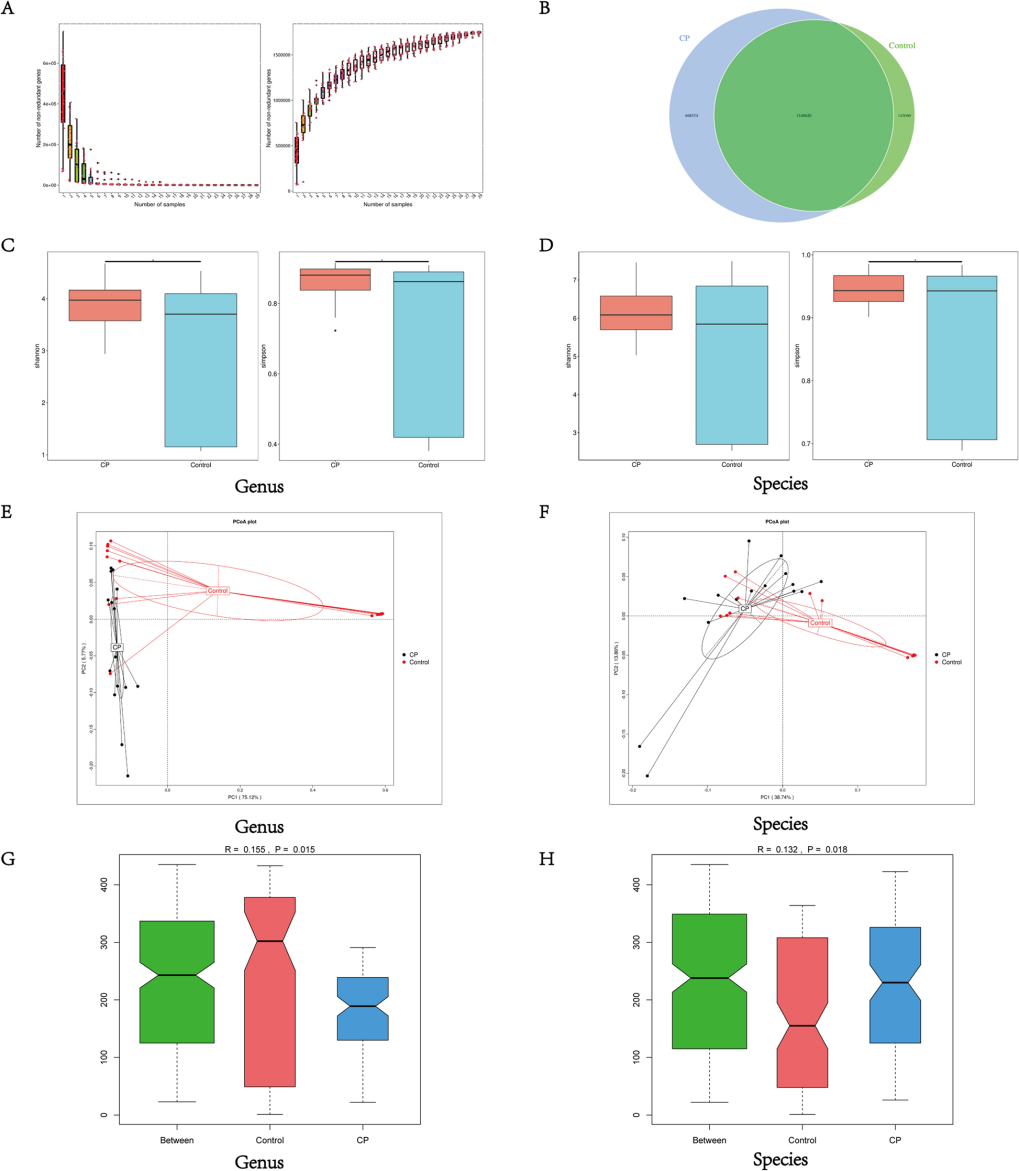
Gene prediction and abundance analysis. **A** Core-Pan gene rarefaction curves. **B** The Venn graph of gene number distribution. **C**-**D** α-diversity analysis (Shannon and Simpson index) at Genus and Species level. **E**-**F** The PCoA plot at Genus and Species level. PCoA plot representing β-diversity in patients with CP and HCs. **G**-**H** The Notched Box plot of Anosim analysis at Genus and Species level. ^*^ indicates *p* < 0.05

Based on the abundance of genes in each sample, we constructed and visualized rarefaction curves for Core and Pan genes to assess whether the sample size utilized for the analysis is sufficient. Core genes represent genes shared by all samples, while pan genes encompass all genes present across the samples. The curve gradually flattens as the sample size increases, suggesting that the current sample size is sufficient to capture the genetic diversity of the microbial community, meeting the needs for further analysis (Fig. 1A). And gene number differences analysis indicated that the number of unique genes in the CP group and HC group was 468,334 and 143,060, respectively (Fig. 1B).

Subsequently, we examined discrepancies of the gut microbiota diversity between patients with CP and HCs. Shannon and the Simpson indexes showed that the α-diversity of the gut microbiota was higher in patients with CP than in HCs at the genus (*p* = 0.028; *p* = 0.021, *t*-test) and species (*p* = 0.063; *p* = 0.021, *t*-test) levels (Fig. 1C, D). Then we plotted the Bray-Curtis-based Principal Coordinates Analysis (PCoA) score map to represent β-diversity (Fig. 1E, F). This map revealed a clear separation between the CP group and the HC group. Anosim analysis was performed to further demonstrate the discrepancies, and the results indicated that the discrepancy between the two groups was significantly greater than the intra-group at the genus (*R* = 0.155, *p* = 0.015) and species levels (*R* = 0.132, *p* = 0.018) (Fig. 1G, H).

**Fig. 2:**
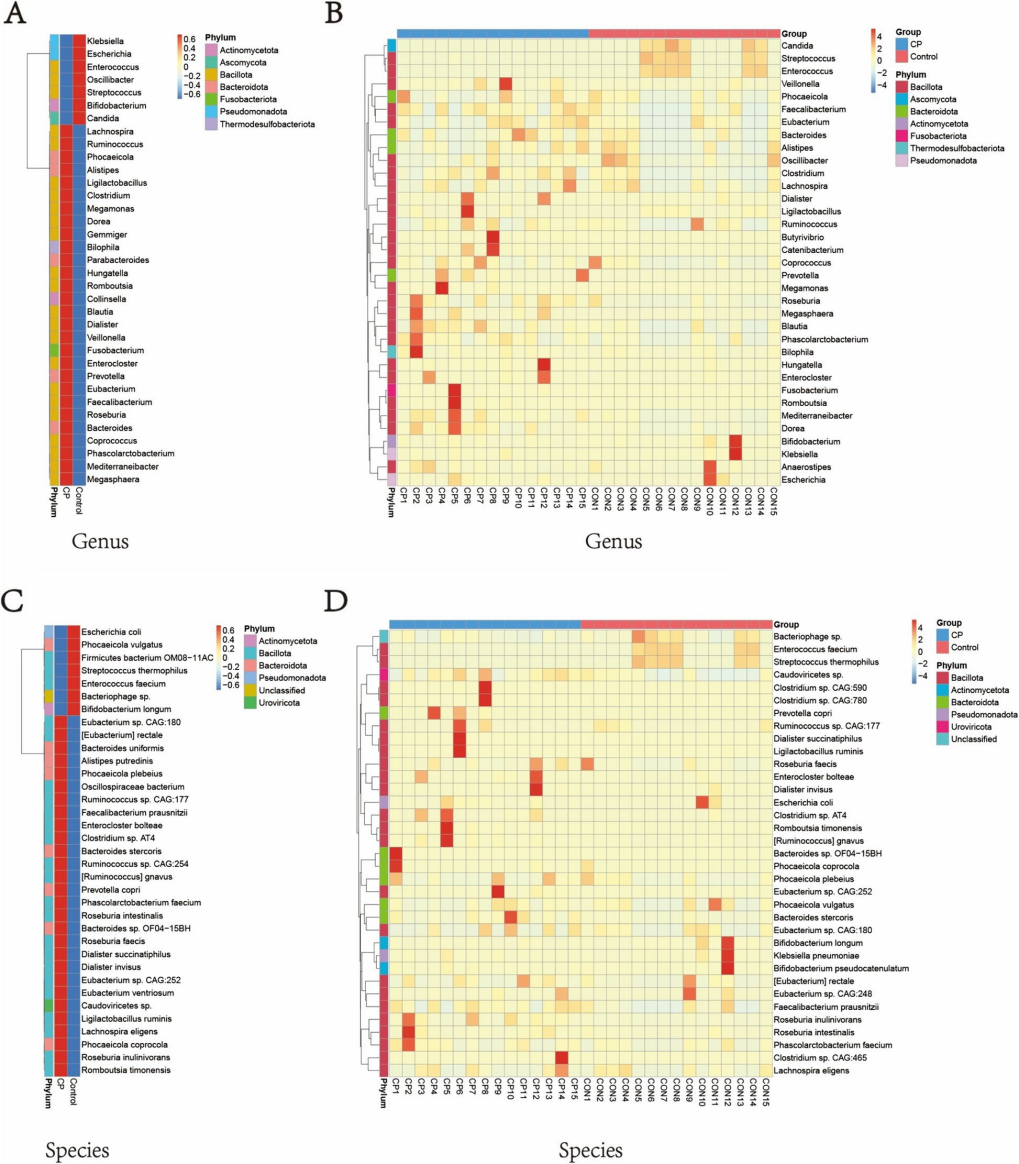
The species abundance clustering heat maps between (**A**, **C**) groups and (**B**, **D**) samples at the (C, D) species and (A, B) genus level

Further, at both the genus and species levels, based on the analysis of species discrepancies between the two groups, we generated species abundance clustering heatmaps for groups and samples (Fig. 2A, B, C, D). As shown in the figures, at both levels, the CP group exhibited a higher relative abundance of microbes primarily within the phyla *Bacillota* and *Bacteroidota*. In contrast, the HC group presented a smaller number of high-abundance microbes compared to the CP group. Across both levels, the variations in microbial relative abundance were predominantly consistent.

**Fig. 3:**
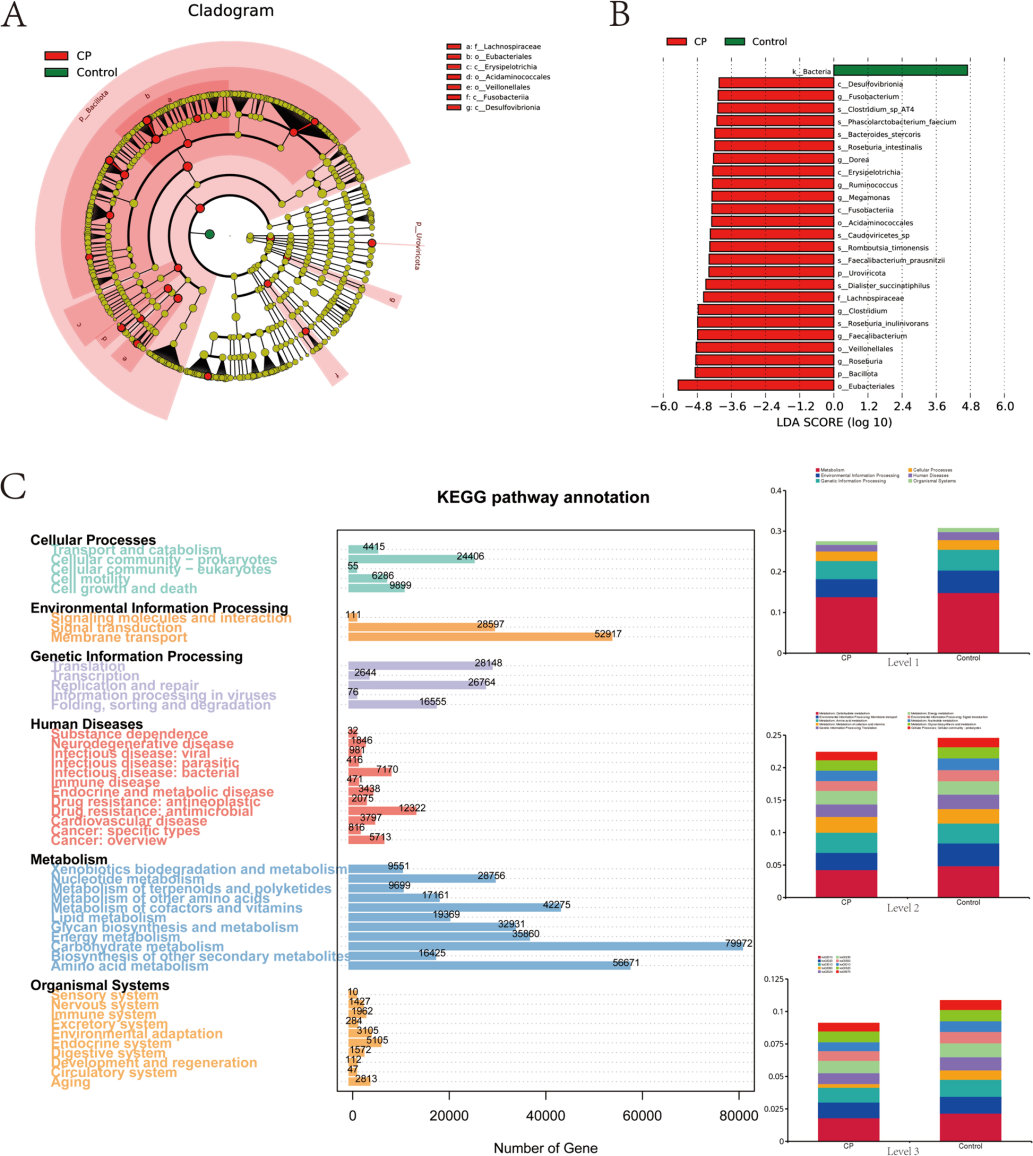
The LEfSe analysis about the species discrepancy and the functional analysis between two groups. **A** The Cladogram of two groups at different levels. **B** The LDA Score distribution map identifying significant biomarkers of difference between the two groups. **C** The KEGG enrichment analysis for differentially expressed unigenes

Furthermore, to identify species with significant differences in abundance between the two groups, we conducted an LDA Effect Size analysis. Subsequently, we created a species evolutionary branching diagram and an LDA value distribution histogram (Fig. 3A, B). As shown in the figure, the CP group demonstrated significant gut microbiota enrichment with genera such as *Fusobacterium, Dorea, Ruminococcus, Megamonas, Clostridium, Faecalibacterium, and Roseburia*. Further analysis at the species level revealed that *Clostridium_sp_AT4, Phascolarctobacterium_faecium, Bacteroides_stercoris, Roseburia_intestinalis, Caudoviricetes_sp, Romboutsia_timonensis, Faecalibacterium_prausnitzii, Dialister_succinatiphilus, and Roseburia_inulinivorans* were also significantly enriched in the CP group. Contrastingly, the broader classification of the kingdom *Bacteria* was found to be depleted. The overall results clearly indicated a significant difference in the composition of gut microbes between the CP group and the HC group.

To characterize the functional variations in the gut microbiome, we conducted metagenomic functional annotation of the Kyoto Encyclopedia of Genes and Genomes (KEGG) module based on unigenes. The results revealed that the most significant variation between the two groups was related to metabolic pathways (Fig. 3C). The most pronounced aspects included Carbohydrate metabolism, Amino acid metabolism, and Metabolism of cofactors and Vitamins. The unique gene content for each aspect was 79,972, 56,671, and 42,275, respectively. Furthermore, our analysis revealed discrepancies between the CP and HC groups at KEGG levels 1 to 3, based on a KEGG pathway analysis of unigenes.

### The difference in fecal metabolism between the CP group and HC group

**Fig. 4:**
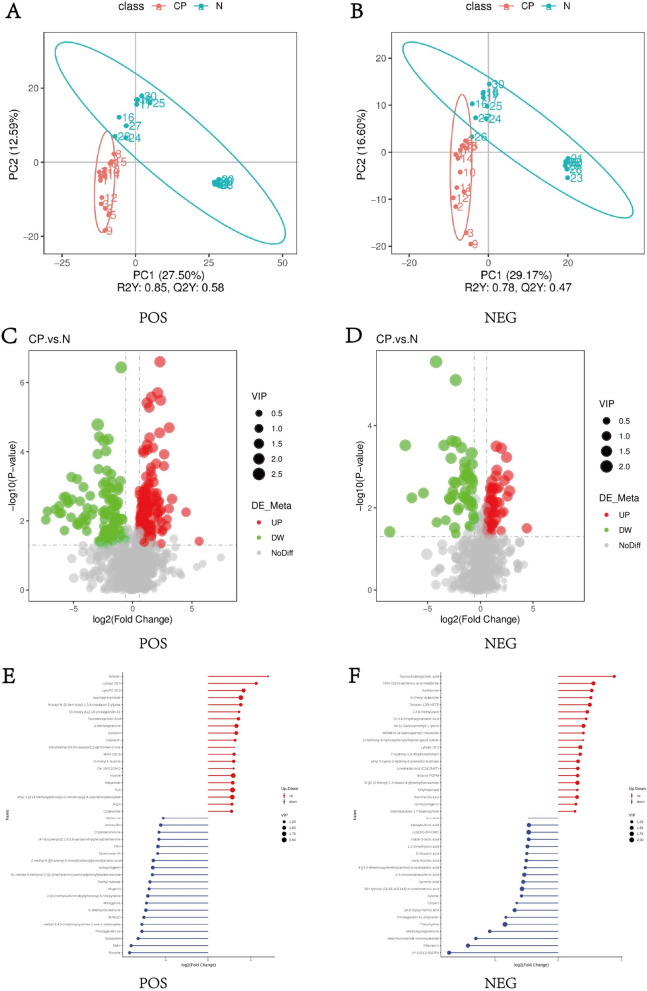
The display of different metabolites. **A**, **B** The Partial Least Squares Discrimination Analysis (PLS-DA) in positive ion mode (POS) and negative ion mode (NEG). **C**, **D** The volcano map of discrepancy metabolites in POS and NEG. **E**, **F** The match map of the top 20 metabolites in POS and NEG

Considering that the genetic composition of the gut microbiota in the CP group and HC group differs significantly, particularly in metabolism, untargeted LC-MS metabolomics were employed to analyze the metabolomic profiles of fecal samples from both groups. Fecal samples were analyzed using LC-MS in positive ion mode (POS) and negative ion mode (NEG), respectively. PLS-DA was employed to build a relationship model between metabolite expression and sample class to differentiate the metabolic profiles among the groups. The model was verified without overfitting and can be applied for further data analysis (Fig. 4A, B).

Subsequently, volcano maps were plotted to depict the overall distribution of differential metabolites between the two groups (Fig. 4C, D). Additionally, to visually identify the up-regulated and down-regulated metabolites with significant differential multiples, we generated match maps of the top 20 metabolites based on the differential metabolites from each group of comparative analyses (Fig. 4E, F). As shown in the diagram, in the positive ion mode, Silibinin, Lysopc 20:0, LysoPC 20:2, and other substances were significantly up-regulated, while Ricinine, EMH, Estazolam, and other substances were markedly down-regulated. However, in the negative ion mode, Tauroursodeoxycholic acid, JWH 018 N-pentanoic acid metabolite, Xanthosine, and other metabolites were clearly up-regulated, whereas (+/−)10(11)-EpDPA, Rifampicin, beta-Nicotinamide mononucleotide, and other metabolites were significantly down-regulated.

**Fig. 5:**
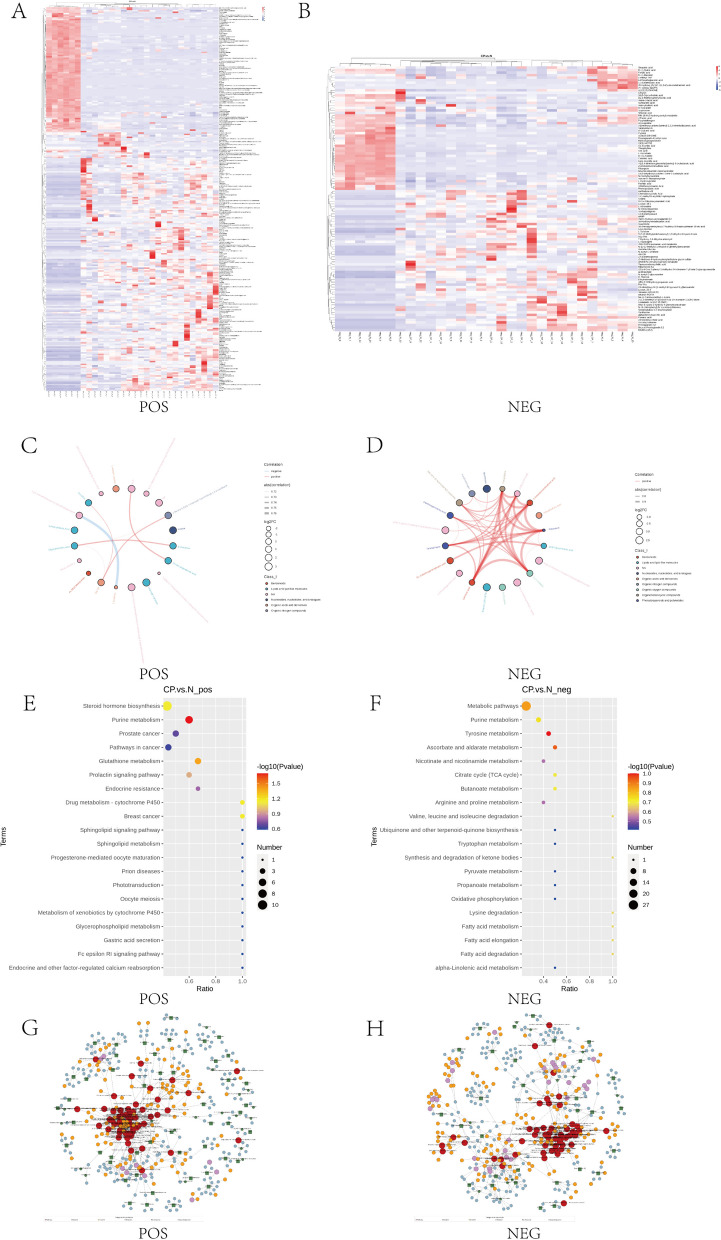
The analysis of different metabolites. **A**, **B** The cluster heatmap of different metabolites in POS and NEG. **C**, **D** The chord diagram of the top 20 different metabolites in POS and NEG. **E**, **F** The bubble diagram of KEGG pathway in POS and NEG. **G**, **H** The gene regulation network map in POS and NEG

Metabolites with similar metabolic patterns (up-regulated or down-regulated) may share similar functions or belong to the same pathway regulating the same metabolic process. Consequently, we conducted cluster analysis focusing on metabolites with comparable metabolic patterns (Fig. 5A, B). To better illustrate the correlation and degree of association between different metabolites in the samples, we generated chord diagrams based on the correlation coefficients of the varying metabolites between the two groups (Fig. 5C, D).

Additionally, bubble plots of the enriched KEGG pathways and regulatory interaction network diagrams for each group of differential metabolites were generated based on the enriched pathways of differential metabolites (Fig. 5E-H).

## Discussion

In this study, metagenomic and metabolomic analyses of fecal samples revealed significant differences in the composition and metabolic profiles of the gut microbiota between patients with CP and HCs. These disparities suggest a potential link between metabolic changes in patients with CP and variations in their gut microbiota.

At the genus level, the gut microbiome, including *Clostridium*, *Roseburia*, and *Faecalibacterium*, was up-regulated in CP group. At the species level, species such as *Roseburia_inulinivorans* and *Dialister_succinatiphilus* were up-regulated. These changes may be associated with various diseases. For example, the *Roseburia* genus, a microorganism primarily producing butyrate in the human body, includes five species: *Roseburia intestinalis*, *Roseburia hominis*, *Roseburia inulinivorans*, *Roseburia faecis*, and *Roseburia cecicola* [15, 16]. Notably, *Roseburia intestinalis* is consistently recognized for its anti-inflammatory properties and its potential to influence the brain through the gut-brain axis [17]. Our findings indicate a notable upregulation of *Roseburia intestinalis* in patients with CP, possibly as a result of the tumor inducing an inflammatory response in the surrounding tissues, leading to the upregulation of several anti-inflammatory factors [18, 19]. Hence, conducting animal experiments to investigate whether *Roseburia intestinalis* can attenuate the inflammatory response in CPs is of significant importance.

Next, to delineate the functional variations of the gut microbiome, we executed metagenomic functional annotations of the KEGG module. Our analysis highlighted metabolism as the principal divergent sector between the CP group and the HC group. Subsequently, we undertook metabolomic analyses which revealed that, in the positive ionization state, 160 metabolites were up-regulated and 107 were down-regulated in CP group relative to HC group. Meanwhile, in the negative ionization state, 56 metabolites were found to be up-regulated and 107 down-regulated in patients with CP. Notably, the augmentation of Silibinin and Lysopc 20:0 in the positive mode was significant in comparison to other metabolites, whereas Tauroursodeoxycholic acid (TUDCA) demonstrated a marked up-regulation in the negative mode.

Silibinin is recognized for its hepatoprotective properties and emerging evidence supports its anti-tumor potential. Yet, contrary to existing findings, our results indicate an increase in silibinin levels in tumor patients. This may reflect the cytotoxic side effect associated with excessive silibinin, as reported in prior research. It is also possible that liver damage in patients with CP, caused by excessive obesity, triggers a protective response in the body [20–22]. Additionally, studies indicate that silibinin may suppress Adrenocorticotropic hormone (ACTH) secretion, potentially explaining the diminished cortisol levels in patient with CP [23].

TUDCA, identified as an up-regulated endoplasmic reticulum (ER) stress inhibitor, has been shown to downregulate apoptotic markers such as caspase-3 and caspase-12 [24]. The downregulation of apoptotic markers may contribute to tumor proliferation, therefore, it is essential to investigate whether this mechanism is implicated in the growth of CPs.

Moreover, ricinine is recognized for its intense toxicity and has been reported to stimulate the nervous system [25]. Recent findings indicate that ricinine can activate the Wnt/β-catenin pathway, which is significantly associated with the progression of CP. Especially in adamantinomatous craniopharyngiomas (ACPs), a prevalent subtype of CP, there is a notable observation of nucleocytoplasmic β-catenin accumulation in most cases, indicative of disease progression [26–28]. Our study, however, observes a downregulation of ricinine in the gut microbiota of CP patients. This downregulation may result from a feedback inhibition mechanism due to protein accumulation in the Wnt/β-catenin pathway, which in turn suppresses ricinine synthesis.

Besides, an increasing body of evidence suggests alterations in host gut microbiota and their metabolites are likely to influence the host’s nutritional status significantly. The association between obesity and CPs is widely acknowledged in current research [29]. It has been hypothesized that CPs may impair the hypothalamic nuclei, such as the ventromedial hypothalamus (VMH) and the hypothalamic paraventricular nucleus (PVN), disrupting the HPA axis, yet targeted treatments for this mechanism remain elusive [30, 31]. Prior studies have established a strong connection between the HPA axis and gut microbes [32–34]. Microbial components like peptidoglycans or their influence on small-molecule cytokines, immunokines, and metabolites within the body can affect the HPA axis. Conversely, the activation of the HPA axis can also induce changes in the gut microbiome [35, 36]. Consequently, our findings imply that the impact of CPs on the HPA axis could be mediated by gut microbiota, presenting potential avenues for therapeutic intervention and management of related complications.

This study recognizes certain limitations. Firstly, we must acknowledge that despite the lack of statistical differences in factors such as age, gender, and BMI between two groups, a detailed analysis of the effect sizes of these potential confounding factors is lacking due to limitations in sample size. Therefore, our preliminary exploration necessitates validation in larger-scale studies with more rigorous control of confounding factors. And conducting multicenter studies could mitigate confounding factors like race, geographical location, and cultural differences. Moreover, this research also did not encompass a comparative analysis of the two craniopharyngioma subtypes, nor validation in animal models, which are essential for future research. Despite these limitations, our research provides valuable preliminary data and illuminates the necessity for further investigation.

## Conclusions

In summary, our research offers substantial evidence of differences in the gut microbiota composition between patients with CP and HCs. Notably, there are also significant differences in metabolic patterns between the two groups. These distinctions may open avenues for developing novel therapeutic strategies.

## Data Availability

Metagenomic raw sequencing data for biosamples have been deposited in SRA database with BioProject ID PRJNA1093500.

## Abbreviations

ACP: Adamantinomatous craniopharyngioma
ACTH: Adrenocorticotropic hormone
BMI: Body mass index
CP: Craniopharyngioma
EEEA: Extended endoscopic endonasal approach
ER: Endoplasmic reticulum
FC: Fold change
GTR: Gross total resection
HC: Healthy controls
HPA axis: Hypothalamic-pituitary-adrenal axis
KEGG: Kyoto encyclopedia of genes and genomes
NEG: Negative ion mode
PCoA: Bray-curtis-based principal coordinates analysis
Pit-NET: Pituitary neuroendocrine tumor
PLS-DA: Partial least squares discriminant analysis
POS: Positive ion mode
PVN: Hypothalamic paraventricular nucleus
TUDCA: Tauroursodeoxycholic acid
UHPLC-MS/MS: Ultra-high-performance liquid chromatography coupled with tandem mass spectrometry
VMH: Ventromedial hypothalamus
